# Occurrence of Deoxynivalenol and Ochratoxin A in Beers and Wines Commercialized in Paraguay

**DOI:** 10.3390/toxins11060308

**Published:** 2019-05-30

**Authors:** Andrea Alejandra Arrúa, Juliana Moura Mendes, Pablo Arrúa, Francisco Paulo Ferreira, Gabriela Caballero, Cinthia Cazal, Man Mohan Kohli, Inocencia Peralta, Gabriela Ulke, Danilo Fernández Ríos

**Affiliations:** 1 Dirección General de Investigación Científica y Tecnológica, Centro Multidisciplinario de Investigaciones Tecnológicas, Universidad Nacional de Asunción, San Lorenzo 1039, Paraguay; jmmarrua@gmail.com (J.M.M.); pdaa88@gmail.com (P.A.); licfranferre10@gmail.com (F.P.F.); gabicmairesse@gmail.com (G.C.); cccazalm86@gmail.com (C.C.); iperalta@rec.una.py (I.P.); 2 Facultad de Ciencias Exactas y Naturales, Departamento de Biotecnología, Universidad Nacional de Asunción, San Lorenzo 1039, Paraguay; gabi.ulke@gmail.com; 3 CámaraParaguaya de Exportadores y Comercializadores de Cereales y Oleaginosas, Asunción 1548, Paraguay; mmkohli@gmail.com

**Keywords:** alcoholic beverages, food safety, mycotoxins, risk

## Abstract

Alcoholic beverages can be contaminated with mycotoxins. Ochratoxin A (OTA) is the most frequently detected mycotoxinin wine and is produced by several species of *Aspergillus.* This mycotoxin is nephrotoxic and carcinogenic. In beer, the most commonly identified mycotoxin is deoxynivalenol (DON). Ingestion of food contaminated with DON has been associated with adverse gastrointestinal effects. Despite the harmful effects of mycotoxins on health, there are no regulations regarding their limits in alcoholic beverages in Paraguay. Here we determine the presence of OTA and DON in wine and beer, respectively. Four commercial brands of wine and twenty-nine brands of craft and industrial beerwere tested by the Agra quant ELISA method. One brand of wine was positive for OTA and seven brands of beer (one of them craft) were positive for DON. The values found for both toxins are below the recommended maximum intake proposed by international standards. Giving the high consumption of these products in the country, regulations and monitoring systems mustbe established to check the maximum levels of mycotoxins allowed in alcoholic beverages.

## 1. Introduction

Alcoholic beverages are commonly consumed all over the world [1,2]. In Paraguay, the annual average consumption is 14.2 liters per person per year. Men arethe largest group of consumers, drinking around 20% more than the average [3]. Several studies have suggested thatthe consumption of some alcoholic beverages such as wine and beer could have both positive and negative effects on human health depending on the amount ingested [4,5,6].

Moderate consumption of wine has been linked to a reduced risk of developing some health conditions, including age-related macular degeneration [4], cardiovascular diseases [7] and diabetic retinopathy [8]. In addition, some studies have also reported on the improvement of some diseases, such as cancer, osteoporosis and neurological problems [5]. Other positive effects correlated to its consumption are lower blood pressure, enhanced longevity anda boost in lipid metabolism [4,5,9,10,11]. The health benefits of moderate consumption of beer are related to its nutritional, nutraceutical, antioxidant, anti-inflammatory, antidiabetic, antibacterial, antiviral, antithrombotic, antiallergenic, antidiarrhea and vasodilator properties. Moreover, it has been associated with hair strengthening, stimulation of lactating glands and prevention of several types of cancer and gastrointestinal diseases [12,13,14]. Furthermore, the reduction of oxidative damage on the DNA of mucosal cells has been proven in rats [15].

The intake of wine and beer can also lead to adverse health effects. This is the case when these products are contaminated with mycotoxins. Mycotoxins are toxic secondary metabolites for humans [16]. They are produced by certain genera of filamentous fungi, especially *Fusarium* and *Aspergillus*. These organisms can contaminate crops throughout their development, processing and shelf life. This makes the contamination risk management of these products extremely complex and difficult [17,18].

Considering that mycotoxins can be present in the final products when the raw material used for their elaboration is contaminated [19], it is important toimplement monitoring systems throughout the production chain that guarantee not only the quality but also the safety of the product [20,21]. In the case of nutraceutical products, extreme care needs to be taken given their potential use in the improvement of human health [22].

The most frequently detected mycotoxin in wine is ochratoxin A (OTA) [23,24,25,26,27,28,29,30]. Reports of OTA in wine have been frequent around the world in recent years [23,26,31,32]. The International Agency for Research on Cancer (IARC) places it in group 2B due to its possible carcinogen effect in humans [33,34]. Dietary exposure to OTA represents a serious health problem and has been associated with several human and animal diseases, including human endemic nephropathies and tumors in the urinary tract, among others [35]. A recent study analyzed samples of wine and grape juice from various countries and found them to reach high levels of OTA contamination. In many cases, these levels exceeded the maximum values established by international standards such as Agência Nacional de Vigilância Sanitária (ANVISA, by its acronyms in Spanish) or the European Union (EU) [36,37]. Moreover, up to 70% of the examined samples were contaminated [23].

Deoxynivalenol (DON) is the most commonly detected toxin in beer [38,39,40]. It is a very stable trichothecene found during the storage and processing of products [18,41]. DON is associated with acute gastrointestinal adverse health effects, such as vomiting (emesis). In the long term, the main effects associated with its consumption are the suppression of weight gain, anorexia, altered nutritional efficiency, hepatotoxicity, dermatological problems and effects on the immune and gastrointestinal systems [42].

In developing countries such as Paraguay, compliance with mycotoxin regulations could guarantee the protection of the population against adverse health effects from exposure to mycotoxins. While regulations concerning aflatoxins in peanuts, corn and milk are in place in Paraguay, those concerning OTA or DON in alcoholic beverages are non-existent. As mycotoxin regulations are deficient, conditions are created in which exposure to toxins can occur above the internationally established levels [36,37]. 

Currently, there are no regulations on the permissible levels of mycotoxins in wine and beer in Mercado Común del Sur (MERCOSUR). In 2011, Brazil proposed the maximum tolerable concentration of 1750 mg kg^−1^ for DON in grains of malted barley (main ingredient of several types of beer) [36]. As for the EU, the limit set in cereals and their derived products is 1750 mg kg^−1^ [37].

In order to establish the proper regulations for mycotoxins in alcoholic beverages, data on the presence of OTA and DON in these products are required. Thus, the aim of this work was to determine the presence or absence of mycotoxins in wine and beer commercialized in the metropolitan area of Asunción in Paraguay.

## 2. Results and Discussion

### 2.1. Ochratoxin Levels in Wine

In order to analyze the OTA levels in wine and grape juice from common brands consumed in Asunción, Paraguay, four brands of wine and one brand of grape juice were selected. All the wines analyzed were red. The results obtained are summarized in Table 1. Only one out of the five samples analyzed was positive for OTA. This sample (brand A) corresponds to a Chilean wine and was the most expensive product studied. It showed an intake risk level of 2.42 ppb on average, exceeding the values established by the EU and ANVISA.The OTA levels of the other samples (75% of the samples) could not be detected with the applied methodology. 

Similar results were observed by Vega et al. when studying Chilean wines [43]. In this study, most of the analyzed samples presented OTA levels around 0.01 ppb in red wines, with only one sample above that value (0.35 ppb). Therefore, the researchers suggested that the incidence of OTA in Chilean wines is very low compared to other wine-producing regions and that Chile is in a privileged situation with the lowest wine incidence of OTA in the world.

Regarding the Argentinian wines, our results were again in agreement with previous findings reported by Ponsone et al. [44]. This study showed that Argentinian wines presented OTA levels between 0.12 and 0.37 ppb in red wines. It is worth noting that higher levels of OTA in Argentinian wines indicate that the geographic origin of the wines probably plays an important role in the presence and levels of this mycotoxin. This is due to the climatic conditions in which the grapes and wine are produced [45]. 

In our study, the differences in the alcoholic grade of the sample analysis did not influence the OTA content. Both the grape juice and most of the different wines had no detectable levels of this toxin. Levels of OTA in Brazilian juice had not been studied before. Contamination with fungi producing ochratoxins is influenced by biotic and abiotic factors affecting the crop, which can also change from year to year. Additionally, factors affect the OTA content during the industrialization process. Thus, it is necessary to puta monitoring system in place that guarantees not only the quality, but also the safety of the product.

A relevant aspect is the monitoring requires evaluation of mycotoxins in the vineyards to avoid the subsequent contamination of the product and to ensure its safety [46]. OTA is an important contaminant of various types of food andthe wine. Their presence has been detected in human fluids, including fetal maternal fluids, but they do not represent a risk to the mother and fetus due to their frequency and level of exposure [47].

### 2.2. Deoxynivalenol Levels in Beer

DON is a very stable mycotoxinin the process of beer production. It is transferred from barley to beer and its concentration increases throughout the production process. In order to analyze the DON levels in beers from common brands consumed in Paraguay, 29 brands of beer were selected. The results obtained are summarized in Table 2.

Almost a quarter (24.13%) of the samples studied were positive for DON, with an average of 1.54 ppb. Compared to previous work done in other regions, the percentage of contamination is low [38]. In 2011, 106 samples of beer collected in different European countries were analyzed, and variable levels of DON were found in 66% of the analyzed samples. In this study, the levels of DON in beer were variable among countries, with the lowest mean value being 0.9 ± 0.9 µgL^−1^ (900 ppb) in the samples from the Czech Republic and the highest being 4.1 ± 3.3 µg L^−1^ (4100 ppb) in the samples from Belgium.

In 2018, another study evaluated the levels of DON in 100 samples of beer selected from the Polish market and 83% of the samples were found to be contaminated [39]. The surveyed samples displayed a great variation in the DON levels, but all were below those established by international regulations, thus indicating that beer is not an important source of mycotoxin intake. In another study carried out in 2017, 1000 samples of craft beer from 47 countries were analyzed and the results showed that the mycotoxin with the highest incidence was DON [48]. This study concluded that it is necessary to set maximum limits of mycotoxins in beer in order to protect consumers from beer with high levels of DON. DON is the most common contaminant in cereals around the world, especially in wheat and barley [49]. Therefore, it is important to monitor the levels of this mycotoxin in cereal derivatives. When performing the analysis of variance by brands, no significant differences were observed between the samples with positive values. The highest value of DON detected was 8.58 ppb. As for the beer presentation (type of packaging), no significant differences were observed; however, the canned beers presented DON contents three times higher in absolute value (3.43 ppb) than those packed in bottles (1.08 ppb).

Regarding the type of beer, no significant differences were found between industrial and craft beers for the analyzed samples. The mean content of DON in industrial beers was 1.88 ppb, while in craft beerit was 1.08 ppb. No significant differences were observed when analyzing samples considering their geographical origin, but it is important to note that domestic beers had the highest levels of DON, with a mean of 2.23 ppb compared to their imported counterparts, with a mean of 0.61 ppb of DON. As for the beer category, there was no significant differences; but in the Golden Ale, the mean DON content was 1.08 ppb, and in the Lager it was 1.88 ppb. Finally, considering the content of alcohol and main ingredient, there were no significant differences in DON contamination. In conclusion, none of the analyzed categories (type of packaging, geographical origin, beer category and craft vs. industrial) had a significant influence on the DON contamination levels.

### 2.3. Estimation of the Daily Intake of OTA and DON in Wine and Beer

The per capita consumption of beer in the Paraguayan population is estimated to be 66.8 liters per person per year with a daily consumption of 0.18 liters based on a mean of 1.70 ppb, equivalent to 1.70 mg kg^−1^ and a body weight of 60 kg [3].

The values found for both OTA in wines and DON in beers are below recommendations set by international standards Table 3. It must be noted that Paraguay does not produce barley, and that all the malt for brewing beer, both industrial and craft, is imported. Presumably, the toxins found in the brands under study come from the barley. In 2011, an analysis of 106 brands of beers collected in Europe determined risk levels of 0.018 mg L^−1^bw, lower than those found in this study of beers (0.0036 mg kg^−1^bw) [38].

It is important to keep in mind that wine and beer are considered to be nutraceutical foods. Whether or not they possess potential pharmaceutical use [50], strict controls must be carried out to not only ensure the quality, but also the safety of the final product. Considering that the regulations that affect food are also applied to nutraceutical foods, it can be assumed that the established limits for mycotoxins should be respected [51].

## 3. Conclusions

This is the first DON and OTA intake risk assessment performed on wine and beer in Paraguay. One-fourth of the wine samples analyzed were positive for OTA and almost the same percentage of the beer samples were positive for DON.Despite the low levels of both mycotoxins, OTA found in wine and DON in beer, the presence of these contaminants represents a call to action for authorities and the industry. Regulations andmonitoring systems mustbe established on the maximum levels of mycotoxins allowed in alcoholic beverages given the high consumption of these products in the country.

## 4. Materials and Methods

### 4.1. Wine

For ochratoxin extraction in wine, 3 mL of wine were pipetted into a test tube and 5.7 mL of 100% methanol was added. The tube was vortexed and mixed for 30 sand the pH value was adjusted in the range of 6.0–8.0 using 1 M NaOH. Subsequently, the determination of ochratoxins in wine and grape juice was carried out using the Agra quant® Ochratoxin Assay kit 2/40 ppb from Romer Labs [52]. Five commercial brands of wine (2 Chilean and 2 Argentinian) and one of grape juice (Brazil) were purchased at random in supermarkets of the metropolitan area of Asunción, Paraguay. Analysis of variance and comparison of means were performed using the Tukey test with a confidence interval of 95%.

### 4.2. Beer

For the extraction of DON in beer, 20 mL of beer and 100 mL of deionized water was added. The sample was vortexed and mixed for 30 s. Once the sample had settled, it was filtered with a #1Whaltman filter and the pH value was adjusted in the range of 6.5–7.5 using 1M NaOH. The extract was diluted in a 1:4 proportion and the determination of DON was made by using the Agra quant® Deoxynivalenol Assay 0.25/5.0 ppm from Romer Labs [53]. Twenty-nine commercial brands of beer were purchased at random; 12 local (3 craft and 9 industrial) and 17 imported (2 Dutch, 3 Argentinian, 1 American, 1 Belgian, 1 Mexican, 1 Chilean, 1 Spanish, 2 Italian, 2 German and 1 English). All industrial beers were purchased at random in supermarkets of the metropolitan area of Asunción, Paraguay. Analysis of variance was performed using the Tukey test with a confidence interval of 95%.

### 4.3. Calculation of OTA and DON Intake Risk

The values were calculated as follows: mycotoxin intake: mg kg^−1^ per day = average consumption of mycotoxin per day expressed in mg kg^−1^, assuming a body weight of 60 kg [49,54,55].

## Figures and Tables

**Table 1:** Ochratoxin A (OTA) intake risk level in wines and grape juice.

Brand	Product	Origin	Cost	OTA ppb
A	Red wine	Chile	Medium	2.42
B	Redwine	Chile	Low	ND
C	Redwine	Argentina	Low	ND
D	Red wine	Argentina	Low	ND
E	Grape juice	Brazil	Low	ND

ND—not detectable. Detection limit: 2 ppb.

**Table 2:** Deoxynivalenol (DON) concentration in beer (mg L^−1^).

Brand	Production	Country	Mean DON ppb	% Alcohol	Category
A	Industrial	Holland	ND	5	Lager
B	Industrial	Paraguay	ND	4.5	Lager
C	Industrial	Argentina	ND	5.2	Bohemian
D	Industrial	Argentina	ND	4.2	Weisse
E	Industrial	Belgium	ND	5	Lager
F	Craft	Paraguay	ND	4.8	Red Ale
G	Industrial	México	ND	4.5	Lager
H	Industrial	USA	ND	4.6	Lager
I	Craft	Paraguay	1.08 ^a^	5.7	Golden Ale
J	Industrial	Paraguay	ND	4.6	Lager
K	Industrial	Paraguay	ND	4.8	Lager
L	Industrial	Paraguay	0.73 ^a^	4.6	Lager
M	Industrial	Paraguay	ND	4.7	Lager
N	Industrial	Paraguay	0.46	4.7	Lager
O	Industrial	Paraguay	8.59 ^a^	4.8	Lager
P	Industrial	Paraguay	0.29 ^a^	5	Lager
Q	Industrial	Argentina	0.77 ^a^	5	Lager
R	Craft	Paraguay	ND	4.8	Pale ale
S	Industrial	Brazil	ND	4.5	Pilsner
T	Industrial	Paraguay	ND	5	Pilsner
U	Industrial	Chile	ND	5.3	Lager
V	Industrial	Spain	ND	4.7	Lager
W	Industrial	Italy	ND	4.6	Lager
X	Industrial	Germany	ND	5.3	Lager
Y	Industrial	Germany	ND	4.9	Lager
Z	Industrial	Germany	ND	5	Lager
AA	Industrial	Italy	ND	5.1	Lager
BB	Industrial	England	ND	5.2	Lager
CC	Industrial	Holland	0.45 ^a^	8.5	Lager

ND—not detectable. Detection limit: 0.25 ppb. Values with a superscript are not significantly different (*p* > 0.05).

**Table 3:** DON levels in wine and beer and consumption risk. Mycotoxin intake: mg kg^−1^ per day = average consumption of mycotoxin per day expressed in mg kg^−^^1^, assuming a body weight of 60 kg.

Mycotoxin	Mean mg kg^−1^	Daily Exposure by Body Weight mg kg^−1^bw
OTA red wine	0.28	ND
DON beer industrial and craft	1.77	0.005
DON craft beer	1.08	0.003
DON industrial beer	1.88	0.006
